# Incidence trends of esophageal squamous cell and adenocarcinoma in Finland in 2000–2021

**DOI:** 10.2340/1651-226X.2025.44097

**Published:** 2025-08-01

**Authors:** Pietari Junkala, Anssi Auvinen

**Affiliations:** a Faculty of Medicine and Health Technology, Tampere University, Tampere, Finland; b Faculty of Social Sciences, Tampere University, Tampere, Finland

**Keywords:** Epidemiology, incidence, esophageal neoplasms, esophageal squamous cell carcinoma, adenocarcinoma

## Abstract

**Background and purpose:**

Esophageal cancer (EC) histological subtypes have contrasting incidence trends according to previous studies. In high-income countries, the incidence of esophageal squamous cell carcinoma (SCC) has decreased, while the incidence of esophageal adenocarcinoma (AC) has increased. This descriptive registry-based study evaluates incidence trends by EC subtype in Finland during 2000–2021.

**Material and methods:**

Data on all EC cases by histological subtype, sex and 10-year age group diagnosed over the period 2000–2021 was obtained from the Finnish Cancer Registry. In total, 6,482 cases (2,604 AC, 2,979 SCC) were observed. Time trends by histology, sex and age group were evaluated with Poisson regression and joinpoint regression.

**Results:**

EC incidence in men increased by an annual percentage change (APC) of 1.3 (95% confidence intervals [CI] 0.8–1.8) while no significant increase was observed in women with APC of -0.1, 95% CI -0.8–0.6). Incidence of AC in men increased with APC of 3.5 (95% CI 2.7–4.2) and by 2.0 (95% CI 0.4–3.6) in women. No consistent trends were observed in SCC incidence although in joinpoint regression, from 2000 to 2006 SCC incidence decreased in men by APC of -6.5 (95% CI -20.3 to -1.1). From 2006 to 2021, rates plateaued with APC of 0.9 (95% CI -0.4 to 7.2). No other joinpoints were identified.

**Interpretation:**

EC incidence increased in Finland during 2000–2021 due to an increase in AC. Incidence of AC increased more than threefold in men, with a lesser increase in women. SCC incidence declined until 2006 and plateaued thereafter.

## Introduction

Esophageal cancer (EC) is globally the 11th most common cancer and the 7th most frequent cause of cancer death. In 2022, EC caused approximately 445,000 deaths globally. EC is typically diagnosed at an advanced stage with a poor prognosis; the 5-year survival is around 20% [1].

The two main histological subtypes of EC are squamous cell carcinoma (SCC) and adenocarcinoma (AC). Although SCC and AC present with similar symptoms, there are some differences in treatment, and the prognosis of AC is slightly better [2–4]. The subtypes are associated with different risk factors. Globally, SCC is the more common subtype, accounting for about 85% of EC. Recent decades have shown opposite incidence trends for the EC subtypes: SCC incidence has decreased, especially in high SCC incidence areas such as Asia, while AC incidence has increased in the European and North American populations with low SCC incidence [5].

Future incidence rate and numbers of AC are predicted to increase in high-income countries, while the incidence of SCC is expected to decrease in most populations [6].

The aim of this study is to characterize the incidence trends of EC and its subtypes, AC and SCC, in Finland from 2000 to 2021.

## Material and methods

### Data source and adjustment

Data on all EC cases diagnosed from 2000 to 2021 was obtained from the Finnish Cancer Registry (FCR). The FCR is a nationwide, population-based cancer registry with an estimated completeness for solid tumors of 96% [7]. The numbers of cases were tabulated by calendar year of diagnosis, histological subtype, sex and 10-year age group. EC cases were defined by ICD-O-3 topography C15, including all subclassifications and divided by histology by ICD-O-3 morphology: SCC (8050–8084), AC (8140–8384), other specified subtype (8011–8046, 8090–8131, 8380–9581) and unspecified subtype (8000–8010). Data on the Finnish population size by calendar year, sex and 10-year age group was obtained from Statistics Finland. Additionally, incidence data on cancers of the gastric cardia, ICD-O-3 topography C16.0, was obtained to assess the potential effect of misclassification of AC.

Due to the Finnish privacy regulations, frequencies of 1–4 cases were not included in the dataset, as they could potentially be individually identifiable. Hence, approximately 10% of EC cases (656 cases) were censored. Multiple imputation was used to assign these frequencies. In total, 100 datasets were created, and the missing values were imputed with predicted counts utilizing Poisson regression. After the multiple imputation, cells with predicted input counts exceeding 4 were replaced by a count of 1–4 (the range of unreleased counts), based on a frequency distribution of the censored counts by histological subtype acquired from the FCR.

### Secondary analysis

Approximately 10% of EC cases (663 cases) were registered with missing or unspecified histology. A secondary analysis was carried out with these cases allocated to either SCC or AC according to a ratio of the registered cases of the subtypes in the given stratum (by sex, age group and either 5- or 6-year calendar period).

### Statistical analysis

Directly age-standardized incidence rates (European standard population) [8] for AC and SCC with 95% confidence intervals (CI) were calculated by calendar year, sex and 10-year age group. Age-adjusted incidence trend (average annual percentage change [APC]) over time was calculated using Poisson regression, with numbers of cases as the outcome and annual population size as the offset variable. The independent variables were sex, age group and calendar year. Changes in time trends of age-standardized incidence rates were assessed with joinpoint regression.

To assess differences in the incidence trends by age and sex, interaction term analyses were conducted. Trend differences between subgroups were evaluated with likelihood ratio tests evaluating the improvement in goodness of fit in nested models with the main effects compared only with those including an interaction term also for sex and year or age group and year. The tests were conducted for all multiple imputation datasets and averages of the p-values are reported.

Statistical analysis and multiple imputation were carried out with StataNow (version 18.5 MP-Parallel Edition, StataCorp LLC). Age-standardized incidence rates were calculated with Excel (Version 16.0, Microsoft 365, Microsoft Corporation). For analysis of changes in time trends, Joinpoint Regression Program (Version 5.3.0.0. Statistical Research and Applications Branch, National Cancer Institute) was used.

## Results

### Patients

A total of 6,482 EC cases were diagnosed in Finland from 2000 to 2021 (Table 1). Of these, 2,979 were SCC (60% of the cases in men) and 2,604 AC (82% in men). Additionally, 236 (4%) cases were of other histological subtypes and 663 (10%) of unspecified subtypes. The male:female ratio was 2.2 for all EC, 1.5 for SCC and 4.4 for AC.

**Table 1:** Number and incidence of esophageal cancers by histological subtype, sex and age group in Finland from 2000 to 2021. (*N* = number of cases, incidence per 100,000 people and 95% confidence interval.)

Subtype	All esophageal cancers		Esophageal squamous cell carcinoma		Esophageal adenocarcinoma		Other specified subtype		Unspecified subtype	

	*N*	Age-standardized incidence	*N*	Age-standardized incidence	*N*	Age-standardized incidence	*N*	Age-standardized incidence	*N*	Age-standardized incidence
**All**	**6,482**	**5.6 (4.9–6.2)**	**2,979**	**2.6 (2.1**–**3.0)**	**2,604**	**2.2 (1.8**–**2.6)**	**236**	**0.1 (0.1**–**0.3)**	**663**	**0.6 (0.4**–**0.8)**
Men*	**4,482**	**8.7 (7.4–9.9)**	**1,790**	**3.5 (2.7**–**4.3)**	**2,125**	**4.0 (3.2**–**4.8)**	**159**	**0.1 (0.1**–**0.5)**	**408**	**0.9 (0.5**–**1.3)**
Women*	**2,000**	**3.0 (2.4–3.6)**	**1,188**	**1.8 (1.3**–**2.3)**	**478**	**0.7 (0.4**–**1.0)**	**78**	**0.1 (0.0**–**0.2)**	**255**	**0.4 (0.2**–**0.6)**
Time period*										
2000–2004	1,149	5.2 (4.5–5.9)	647	3.0 (2.4–3.5)	332	1.4 (1.1–1.8)	26	0.2 (0.0–0.3)	144	0.7 (0.4–0.9)
2005–2009	1,285	5.2 (4.6–5.9)	586	2.4 (1.9–2.8)	489	2.0 (1.6–2.4)	40	0.2 (0.0–0.3)	171	0.7 (0.5–1.0)
2010–2014	1,492	5.5 (4.9–6.2)	663	2.5 (2.0–2.9)	635	2.3 (1.9–2.7)	58	0.2 (0.1–0.4)	136	0.5 (0.3–0.7)
2015–2019	1,811	6.1 (5.4–6.7)	767	2.6 (2.2–3.0)	798	2.7 (2.3–3.1)	71	0.2 (0.1–0.4)	175	0.6 (0.4–0.8)
2020–2021	744	6.0 (5.4–6.6)	315	2.6 (2.2–3.0)	350	2.9 (2.5–3.3)	42	0.3 (0.2–0.4)	38	0.3 (0.1–0.4)
Age group*		Age-specific incidence		Age-specific incidence		Age-specific incidence		Age-specific incidence		Age-specific incidence
Under 50	267	0.4 (0.3–0.4)	97	0.1 (0.1–0.2)	143	0.2 (0.2–0.2)	7	0.01 (0.003–0.02)	19	0.03 (0.02–0.04)
50–59	985	5.8 (5.5–6.2)	423	2.5 (2.3–2.7)	457	2.7 (2.4–2.9)	41	0.2 (0.2–0.3)	64	0.4 (0.3–0.5)
60–69	1,983	14.0 (13.4–14.7)	961	6.8 (6.3–7.2)	805	5.7 (5.3–6.1)	58	0.4 (0.3–0.5)	159	1.2 (1.0–1.3)
70–79	1,960	20.1 (19.2–21.0)	880	9.1 (8.5–9.7)	801	8.3 (7.7–8.8)	86	0.9 (0.7–1.1)	193	1.9 (1.6–2.1)
Over 80	1,286	23 (21.8–24.3)	617	11.1 (10.2–12.0)	397	7.0 (6.3–7.7)	44	0.8 (0.5–1)	228	4.1 (3.6–4.6)

* Numbers are approximations because of censoring.

Values for the whole time period and across all age groups are highlighted in **bold**.

### Incidence rates

From 2000 to 2021, the age-standardized incidence rate (per 100,000 persons) of EC was 5.6 (95% CI 4.9–6.2), of which SCC was 2.6 (95% CI 2.1–3.0) and AC 2.2 (95% CI 1.8–2.6). Men had a higher incidence, 8.7 (95% CI 7.4–9.9) for all EC, with 3.5 (95% CI 2.7–4.3) for SCC and 4.0 (95% CI 3.2 to 4.8) for AC, while the incidence was lower in women, 3.0 (95% CI 2.4 to 3.6) for EC overall, 1.8 (95% CI 1.3–2.3) for SCC and 0.7 (95% CI 0.4–1.0) for AC.

### Incidence trends

The age-adjusted incidence of overall EC increased by an APC of 0.9 (95% CI 0.5–1.3) from 2000 to 2021. In men, the APC was 1.3 (95% CI 0.8–1.8) and among women -0.1 (95% CI -0.8 to 0.6) (Table 2).

**Table 2:** Incidence trends of esophageal cancers by histological subtype, sex and age group in Finland from 2000 to 2021. Annual percentage changes with 95% confidence intervals presented.

Subtype	All esophageal cancers	Esophageal squamous cell carcinoma	Esophageal adenocarcinoma
**Men**	**1.3 (0.8–1.8)**	**–0.4 (–1.2**–**0.3)**	**3.5 (2.7–4.2)**
Age group			
Under 50	3.3 (0.6–6.0)	1.6 (–3.3–6.8)	5.3 (2.0–8.8)
50–59	–0.9 (–2.0 to 0.3)	–3.7 (–5.4 to –1.9)	1.4 (–0.2 to 3.0)
60–69	2.4 (1.5–3.2)	0.9 (–0.4 to 2.1)	4.3 (3.0–5.7)
70–79	2.5 (1.6–3.4)	1.5 (0.2–2.9)	4.3 (3.0–5.6)
Over 80	–0.8 (–2.1 to 0.5)	–4.3 (–6.3 to –2.2)	2.8 (0.7–5.0)
**Women**	**–0.1 (–0.8 to 0.6)**	**–0.6 (–1.5 to 0.3)**	**2.0 (0.4–3.6)**
Age group			
Under 50	4.1 (–0.8 to 9.2)	3.4 (–3.2 to 10.5)	6.0 (–2.3 to 14.9)
50–59	1.7 (–0.8 to 4.3)	–0.2 (–3.5 to 3.1)	4.1 (–0.7 to 9.0)
60–69	1.7 (0.1–3.4)	1.4 (–0.5 to 3.4)	1.7 (–1.9 to 5.4)
70–79	–0.2 (–1.5 to 1.0)	–0.2 (–1.8 to 1.4)	1.2 (–1.3 to 3.7)
Over 80	–2.1 (–3.2 to –0.9)	–2.9 (–4.3 to –1.3)	1.6 (–1.4 to 4.7)

Values across all age groups are highlighted in bold

**Figure 1:**
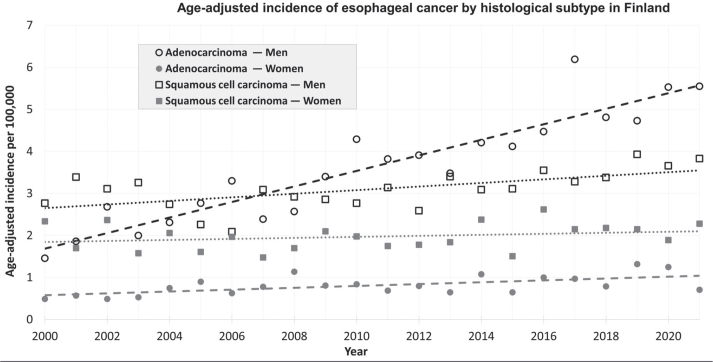
Incidence of esophageal cancer in Finland from 2000 to 2021 by histological subtype, trend line fitted with simple linear regression.

The age-adjusted incidence of AC increased by an APC of 3.2 (95% CI 2.5–3.8) for both sexes combined. There was an increase in both men (APC 3.5, 95% CI 2.7–4.2) and women (2.0, 95% CI 0.4–3.6) (Figure 1). The increasing trend of AC was statistically significantly higher in men than in women (likelihood ratio test *p* = 0.03).

The age-adjusted incidence of SCC showed a non-significant decrease with an APC of -0.5 (95% CI -1.1 to 0.1) for both sexes combined. Comparable APC decreases were seen in men (-0.4, 95% CI -1.2 to 0.3) and women (-0.6, 95% CI -1.5 to 0.3). No significant difference was found in incidence trends of SCC between men and women (*p* = 0.28).

Age-adjusted incidence (per 100,000 persons) of gastric cardia cancer increased from 2000 to 2021 in men from 3.3 to 4.7 and in women from 1.6 to 1.8. Incidence of gastric cardia cancer is presented in Supplementary Appendix A.

Incidence trends of overall EC, SCC and AC differed statistically significantly (*p* < 0.05) by age group, although no consistent pattern by age could be established. The overall EC incidence increased only slightly or even decreased in the age group 50–59 years and in those aged over 80 years. Among these age groups, AC increased only slightly and the decrease of SCC was most pronounced. As an exception to the decrease of SCC, incidence increased in men aged 70–79 years. AC incidence increased most strongly in men aged under 50, 60–69 and 70–79 years. Statistically significant age-specific incidence trends could not be identified among women.

### Joinpoint analysis

**Figure 2:**
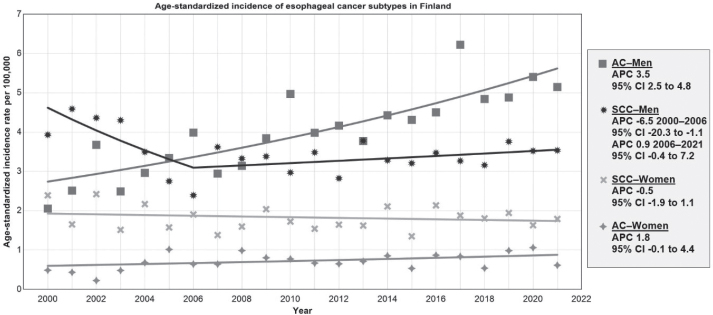
Trends of esophageal cancer incidence in Finland from 2000 to 2021 by histological subtype, age-standardized to the European standard population. APC: Annual percentage change; CI: Confidence interval; AC: adenocarcinoma; SCC: squamous cell carcinoma.

A constant trend with no joinpoints identified was observed for both sexes combined and separately for EC and AC (Figure 2).

Joinpoints were detected for SCC for both sexes combined and among men. From 2000 to 2006, the age-standardized SCC incidence decreased in the whole population with an APC of -5.2 (95% CI -15.3 to -1.3) and in men by -6.5 (95% CI -20.3 to -1.1). From 2006 to 2021, the rates plateaued with an APC of 0.8 (95% CI -0.1 to 4.2) for both sexes combined and in men by an APC of 0.9 (95% CI -0.4 to 7.2). All age-standardized incidence rate trends from joinpoint regression are reported in Supplementary Appendix B.

### Secondary results

Analysis of the imputed datasets where cases with unspecified histology were allocated to either AC or SCC showed results similar to the primary analysis. The increase in incidence of AC was slightly lower after imputation with an overall APC of 2.8 (95% CI 2.1 to 3.4), for men 3.1 (95% CI 2.4 to 3.8) and for women 1.6 (95% CI 0.1 to 3.1). The decrease in incidence of SCC was clearer with an overall APC of -1.0 (95% CI -1.5 to -0.4), for men -0.9 (95% CI -1.6 to -0.2) and for women -1.0 (95% CI -1.8 to -0.1). Joinpoint analysis gave results similar to the primary results, with the only joinpoints identified in SCC incidence in 2006 for both sexes combined and for men. All secondary results are reported in Supplementary Appendix C.

## Discussion and conclusion

From 2000 to 2021, the age-adjusted incidence of EC among men has approximately doubled in Finland from 5.1 to 9.8 per 100,000. The increase is due to a strong increase in AC incidence. Among women, the incidence of EC was lower and showed no significant trends, although the incidence of AC appeared to increase modestly. The incidence of SCC declined from 2000 to 2006 and later stabilized or slightly decreased from 2006 to 2021. The incidence of AC exceeded that of SCC in 2009 in men. In women, SCC has remained the most common subtype. Although these trends varied by age group, no consistent age-related pattern was identified.

The results from the secondary analyses, where cases with unknown histology were allocated to either AC or SCC, were comparable to the primary results. The slower increase of AC and clearer decrease of SCC in the secondary analysis can be explained by most cases of unknown histology being diagnosed in the earlier part of the study period, when SCC was more common than AC. The APCs of the secondary analysis were, however, within the 95% CI of the primary results.

### Esophageal cancer

Incidence of EC in Finland is comparable to other European and Nordic nations, and also share a similar male to female ratio [9]. For over a decade, the rates of EC in Finland have been higher than in Sweden, slightly lower than in Norway and substantially lower than in Denmark [10].

Similar EC incidence trends have been identified in other high-income countries, while clear declines in incidence have been reported in some low-income countries [9].

To improve the comparability between the Finnish and Swedish results, we applied a similar age-standardization as in previous Swedish studies (Swedish population in 2000) [11]. This had no clear effect on the incidence trends or on joinpoint regression. Our results are therefore consistent with those reported from Sweden and are not affected by the differences in age-standardization.

### Esophageal adenocarcinoma

AC is the dominant EC subtype in many high-income populations. The highest rates of AC have been reported from Australia, North America and Northern and Western Europe [12]. AC incidence in Finland is comparable to these populations [9, 12]. Globally, the number of AC cases is predicted to nearly double from 2020 to 2040 [12].

Similar increases in AC incidence have been reported from several highly developed nations [9]. Incidence trends of AC among men in Sweden have been largely similar (APC 1.7 95% CI 0.2 to 3.3) in 2001–2020, although in our study, the increase was more pronounced, while in Sweden the incidence has increased more clearly among women (APC 3.9 95% CI 3.4–4.5) in 1970–2020 [11]. A Czech registry study found increases in AC incidence in 1984–2017 comparable to our results (APC 5.1 95% CI 4.6–5.7) [13]. The SEER registries in the United States have reported a possible stabilization of AC incidence since 2006 (APC -0.4 *p* > 0.05), preceded by a major increase in the previous decades [14].

For AC, the major risk factors include gastroesophageal reflux disease (GERD), obesity and smoking, while a reduced risk has been associated with *Helicobacter pylori* infection [1]. Compared to other obesity-related cancers, the association between obesity and AC seems to be stronger; with a 50% increase in relative risk (RR 1.5, 95% CI 1.3–1.7) with each 5-unit increase of the body mass index [15]. Obesity also increases the risk for GERD, the main risk factor for Barrett’s esophagus, the precursor metaplasia for AC [1, 15].

Simultaneously to the increase of AC incidence, obesity has increased in European countries including Finland [16, 17]. Contrasting with the AC increase, GERD prevalence in Finland appears to have decreased in the past decades, based on data from the Global Burden of disease study 2019 [18]. In parallel, the prevalence of *Helicobacter pylori* infection, a protective factor for AC, has decreased [19].

The increase of AC has widely been attributed to the ‘obesity epidemic’ and increasing rates of GERD in high human developmental index countries. This explanation has been questioned, as the rise in obesity does not match the AC trends in timing or magnitude. Some countries such as Australia or Denmark have reported clear increases in AC incidence, despite only modest increases in obesity prevalence. It has been postulated that the increase could be caused by an unidentified, strong and highly prevalent environmental factor [5].

The notable male predominance of AC incidence remains unexplained, as it is unlikely to be explained by risk factors such as more severe reflux in men and a protective role of estrogenic exposures [20].

AC can be difficult to differentiate from AC of the gastric cardia. It has been recommended that epidemiological studies should investigate all ACs near the esophagogastric junction rather than AC alone [21, 22]. To evaluate if the increase in AC incidence can be explained by misclassification of cardia AC, we obtained incidence rates of cardia cancers from the FCR. The age-adjusted rates of cardia cancer have increased concurrently with AC (Supplementary Appendix A). Thus, it does not appear that the increase of AC is due to misclassification of cardia ACs.

### Esophageal squamous cell carcinoma

The major risk factors for SCC are smoking and alcohol use, as well as drinking hot beverages or untreated water, together with low consumption of fruits and vegetables, opium consumption and air pollution. The highest rates of SCC have been reported in the so-called EC belt in Eastern and South-Central Asia as well as in sub-Saharan Africa [12].

Incidence of SCC has remained mostly stable in Europe, with slight increases in some countries and decreases in others. Incidence rate of SCC in Finland is comparable to several other European populations, especially to the Nordic countries [9, 23]. While a Norwegian study from 2001 to 2021 reported fewer cases of SCC than AC, we found slightly more SCC than AC [24].

Men in Sweden and Finland have had similar SCC incidence trends with a clear decrease before 2007 (Sweden 1988–2007; APC -2.80 95% CI -3.0 to -2.6) and 2006 respectively, followed by a levelling-off (Sweden 2007–2020; APC -1.0 95% CI -2.3 to 0.3) [11]. Similarly in the Czech Republic, a plateau of SCC incidence in men (APC 0.3 95% CI -0.3 to 0.8) in 1994–2017 has been reported but with a clear increase of SCC in women (APC 4.5 95% CI 3.6 to 5.4) in 1984–2017 [13] In the United States, a decline in SCC incidence (APC -2.80 95% CI -3.0 to -2.6) has been reported from 2000 to 2018 [14].

The decrease of SCC incidence has been largely attributed to declining smoking and alcohol consumption [23]. Alcohol and tobacco have a synergistic effect on the risk of SCC. Thus, a simultaneous reduction in both can be expected to result in a strong decrease in incidence [25]. The sex differences for SCC incidence can largely be attributed to differences in smoking and alcohol use [20].

### Validity

To strengthen the validity of the study, multiple imputation was employed to compensate for missing data and secondary analyses carried out including cases of unknown histology. Due to privacy regulation, stratum-specific counts one to four were censored in the data obtained, which could introduce error, chiefly misclassification. To minimize the potential error, numbers of cases in the range 1–4 were imputed using histology-specific frequencies of EC.

As joinpoint regression lacks the features for analyzing and pooling multiple datasets, we evaluated possible effects on the analysis by replacing censored counts with either the smallest (one) or the largest possible value (four). Results of these analyses showed no additional joinpoints or other substantial differences compared with the main analysis.

Based on a validation study with data from 1990 to 2014, the completeness of FCR was estimated as 78% for EC. A low completeness of reporting could materially affect the results, although it is unlikely that the histological type of the cases missing from the registry would substantially differ from the registered cases [26].

## Conclusion

The incidence of EC in Finland has increased slightly from 2000 to 2021, with clear and contrasting changes in AC and SCC. The incidence of AC has increased more than threefold in men, with only a very slight increase in women. The incidence of SCC has decreased in both sexes, more strongly in men.

## Supplementary Material

Incidence trends of esophageal squamous cell and adenocarcinoma in Finland in 2000–2021

## Data Availability

The study data is available from the corresponding author on request.
