# Supplementary material for: Incidence trends of esophageal squamous cell and adenocarcinoma in Finland in 2000–2021
Source: Acta Oncol. 2025 Aug 1;64:44097. doi: 10.2340/1651-226X.2025.44097 (PMC12330605; doi:10.2340/1651-226X.2025.44097)
Supplement: Incidence trends of esophageal squamous cell and adenocarcinoma in Finland in 2000–2021 [file AO-64-44097-s1.pdf]

Supplementary material has been published as submitted. It has not been copyedited, or typeset by Acta Oncologica

SUPPLEMENTARY APPENDIX

Appendix A. Incidence of esophageal cancer subtypes and gastric cardia cancer in Finland from 2000 to 2021.

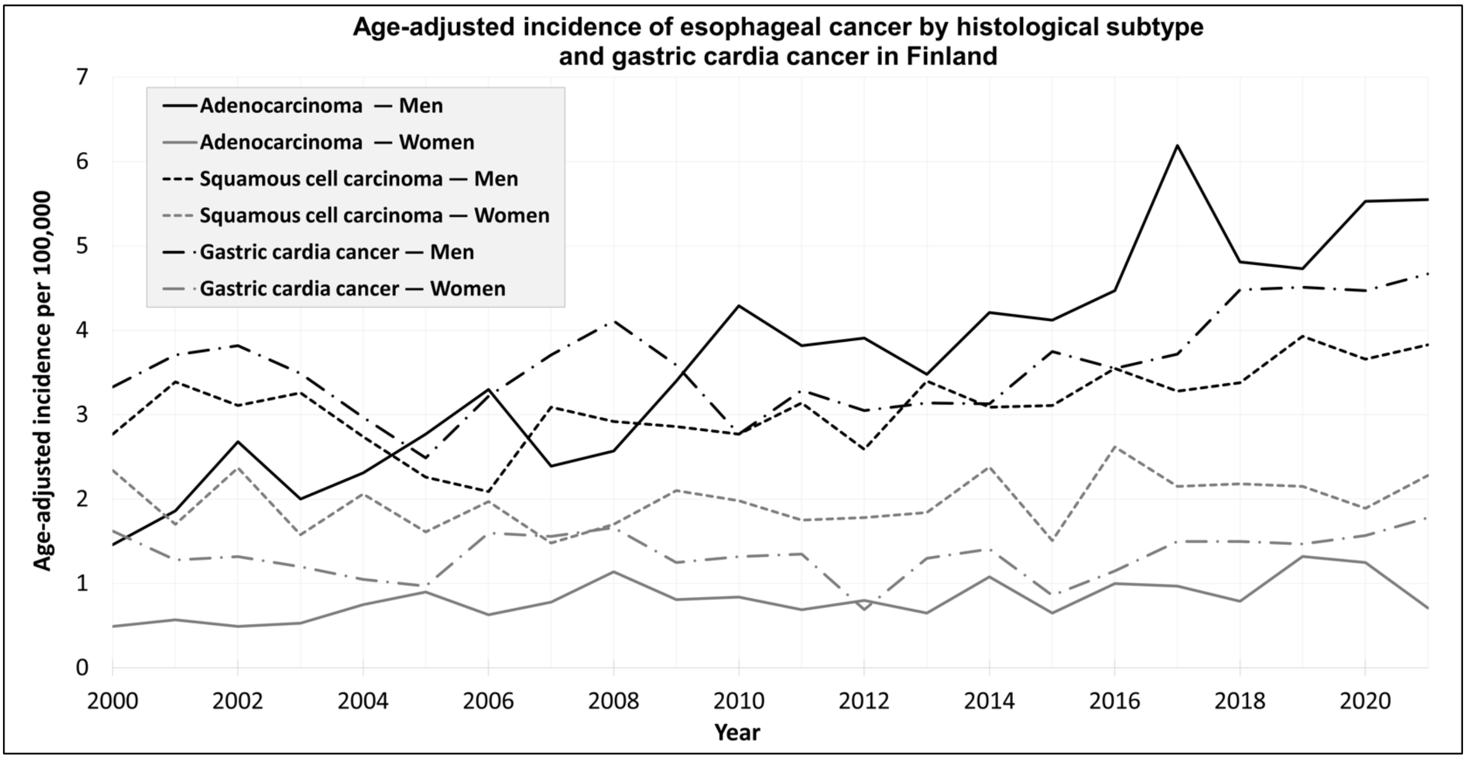

Appendix B. Joinpoint regression age-standardized incidence trends of esophageal cancers in Finland from 2000 to 2021, (APC = Annual percentage change, CI = Confidence interval, age-standardization based on the European standard population)

	All esophageal cancers	Esophageal squamous cell carcinoma	Esophageal adenocarcinoma
	APC (95% CI)		
All	1.0 (0.5 to 1.6)	2000 to 2006: -5.2 (-15.3 to -1.3)<br>2006 to 2021: 0.8 (-0.1 to 4.2)<br>2000 to 2021: -0.9 (-1.7 to 0.1)	3.6 (2.5 to 5.0)
Male	1.3 (0.6 to 2.0)	2000 to 2006: -6.5 (-20.3 to -1.1)<br>2006 to 2021: 0.9 (-0.4 to 7.2)<br>2000 to 2021: -1.2 (-2.3 to 0.2)	3.5 (2.5 to 4.8)
Female	-0.2 (-1.2 to 1.0)	-0.5 (-1.9 to 1.1)	1.8 (-0.1 to 4.4)

Appendix C. Secondary results from datasets where cases of unspecified histology were allocated to either AC or SCC in Finland from 2000 to 2021. (N = number of cases, APC = Annual percentage change, CI = Confidence interval, age-standardization based on the European standard population)

	Esophageal squamous cell carcinoma			Esophageal adenocarcinoma		
	N	Age-standardized incidence (95% CI)	APC (95% CI)	N	Age-standardized incidence (95% CI)	APC (95% CI)
All	3357	2.9 (2.5 to 3.4)	-1.0 (-1.5 to -0.4)	2887	2.4 (2.0 to 2.8)	2.8 (2.1 to 3.4)
Men	1980	3.9 (3.1 to 4.7)	-0.9 (-1.6 to -0.2)	2342	4.5 (3.6 to 5.3)	3.1 (2.4 to 3.8)
Women	1377	2.1 (1.6 to 2.6)	-1.0 (-1.8 to -0.1)	545	0.8 (0.5 to 1.1)	1.6 (0.1 to 3.1)
Age group		Age-specific incidence (95% CI)			Age-specific incidence (95% CI)	
Under 50	104	0.2 (0.1 to 0.2)	1.6 (-2.2 to 5.6)	155	0.2 (0.2 to 0.2)	4.7 (1.7 to 7.9)
50 – 59	454	2.7 (2.5 to 2.9)	-3.0 (-4.4. to -1.5)	491	2.9 (2.6 to 3.2)	1.6 (0.2 to 3.1)
60 – 69	1051	7.4 (7.0 to 7.9)	0.8 (-0.2 to 1.8)	874	6.2 (5.8 to 6.6)	3.7 (2.5 to 4.9)
70 – 79	983	10.0 (9.4 to 10.7)	0.1 (-0.8 to 1.1)	890	9.1 (8.5 to 9.7)	3.0 (1.9 to 4.1)
Over 80	766	13.8 (12.9 to 14.8)	-3.7 (-4.7 to -2.6)	476	8.4 (7.6 to 9.2)	1.7 (0.2 to 3.4)
